# Stress-induced IL-6 response patterns amplify the link between daily negative affect and later depressive symptoms during bereavement

**DOI:** 10.1016/j.bbi.2026.106774

**Published:** 2026-04-20

**Authors:** E. Lydia Wu-Chung, Daniel L. Argueta, Robert Suchting, Ryan L. Brown, Michelle A. Chen, Angie S. LeRoy, Kyle W. Murdock, Cobi J. Heijnen, Christopher P. Fagundes

**Affiliations:** a University of Pittsburgh, Department of Psychology, USA; b Rice University, Department of Psychological Sciences, USA; c University of Texas Health Science Center at Houston, Psychiatry and Behavioral Sciences, USA; d Texas Tech University, Department of Human Development and Family Sciences, USA; e Rice University, Institute for Health Resilience and Innovation, USA; f Texas Christian University, Department of Psychology and Karyn Purvis Institute of Child Development, USA; g University of North Florida, Department of Psychological and Brain Sciences, USA; h Pennsylvania State University, Department of Biobehavioral Health, USA

**Keywords:** Inflammation, Stress reactivity, Depressive symptoms, Widowhood, Daily negative affect, Stressful life event, IL-6, Emotion, Trier Social Stress Test, Depression

## Abstract

Depressive symptoms frequently follow exposure to major life stressors, such as spousal bereavement. However, long-term mental well-being varies considerably. Previously, daily negative affect significantly predicted future depressive symptoms. Higher daily negative affect may signify worse long-term mental health for individuals who also show enhanced inflammatory responses to acute stress, as heightened physiological reactivity to acute stress has been linked with increased susceptibility to depression. Among widow(er)s, we examined whether stress-induced inflammatory responses altered the magnitude of the association between daily negative affect and future depressive symptoms. In exploratory analyses, we examined whether these patterns differed between bereaved and nonbereaved adults. Participants (143 bereaved, 69 nonbereaved) completed the *Trier Social Stress Test (TSST)* and a 7-day affect survey at baseline (4 months post-loss). Subjects self-reported depressive symptoms at baseline and at an 8-month follow-up. Stress-induced IL-6 responses (IL-6 slope) were characterized as the change in serum IL-6 across 3 timepoints (pre-TSST, 45-min post-TSST, 120-min post-TSST). SEM path analysis was used to test hypotheses. Among widow(er)s, we observed a significant negative affect × IL-6 slope effect on future depressive symptoms, after controlling for baseline depressive symptoms and covariates. Widow (er)s with larger stress-induced IL-6 increases showed the strongest, positive association between negative affect and future depressive symptoms. This pattern was not observed in nonbereaved adults. Findings suggest that more daily negative affect in conjunction with heightened stress-induced IL-6 activity may forecast future increases in depressive symptoms. The study identifies possible psychobiological patterns indicative of future maladjustment to stressful life events.

## 1. Introduction

Stressful life events, such as spousal bereavement, are one of the strongest predictors of impending depression. Indeed, among bereaved spouses, mean prevalence rates of major depressive disorder (MDD) reach as high as 38% within the first month post-loss (Kristiansen et al., 2019). Although depressive symptoms normatively increase during early bereavement, symptoms typically subside within 12 months post-loss (Kristiansen et al., 2019; Bonanno et al., 2002). Yet, a small minority experiences persistently elevated depressive symptoms lasting beyond the first year (Kristiansen et al., 2019; Bonanno et al., 2002). Given that depression prognosis and the risk of psychiatric comorbidities and suicide increase the longer depressive symptoms persist (Klein and Kotov, 2016), directing individualized interventions early to those most vulnerable may reduce the burden of depression. After bereavement, widow(er)s face a wide range of stressors, including normal daily hassles, loss-oriented stressors (e.g., grief processing), and restoration-oriented stressors (e.g., adjusting to new social roles, taking on new responsibilities previously performed by the deceased partner) (Stroebe and Schut, 1999). The enduring effects of stressful life events on disease risk depend not only on the duration of the stressor but on the affective sequelae and stress-elicited physiological consequences that persist after the event (Cohen et al., 2019). In other words, one’s emotional and physiological response patterns to acute, innocuous events in their life may be just as, if not more important, to future well-being than the initial major life event itself. The present study explored affective and stress-induced inflammatory patterns as possible predictors of future depressive symptoms following recent spousal bereavement.

Affective patterns may yield valuable insights about health and adaptation in the face of chronic stress. Distinct from stress which pertains to the degree of challenge that exceeds one’s available resources, affect reflects the emotions and mood state that may accompany a given experience (Lazarus, 2006). Outside the context of stressful life events, depression typically presents with an affective profile characterized by high negative affect and low positive affect (Watson et al., 1988). Negative affect in daily life not only distinguishes between depressed and nondepressed individuals, it also associates with future symptom severity among depressed individuals after accounting for baseline symptoms (Cohen et al., 2005; Panaite et al., 2020). However, experiencing negative emotion may be appropriate and adaptive in certain contexts (Coifman et al., 2016), serving survival functions that prompt the individual to confront, escape, or seek help in threatening situations (Nesse and Ellsworth, 2009). According to the dual process model of coping with bereavement, healthy psychological adjustment necessitates the experience of grief and related negative feelings (Stroebe and Schut, 1999; Stroebe and Schut, 2010). Affective profiles differentiate resilient individuals (e.g., low levels of psychiatric symptoms) from maladjusted individuals (e.g., high levels of psychiatric symptoms), such that maladjusted individuals tend to experience one affect dimension over the other (e.g., more negative affect and less positive affect), whereas, resilient individuals experience both positive and negative affect independently of each other (e.g., more negative affect and more positive affect), regardless of concurrent stress (Coifman et al., 2007). Taken together, findings suggest that while higher negative affect is generally associated with worse depression prognosis, this pattern may be more nuanced in chronic stress contexts such as bereavement and may not characterize all individuals.

Stress reactivity patterns may represent individual differences in depressive symptom severity, as abnormalities in the stress response system are observed in those with greater depressive symptoms (Fagundes et al., 2013; Fiksdal et al., 2019). Autonomic, neuroendocrine, and immunological responses to stress are evolutionarily adaptive, equipping the body with the cognitive, metabolic, and behavioral resources to confront and/or neutralize a threatening stimulus. The biological stress response is adaptive when the magnitude of the response matches the level of threat and when the body promptly returns to homeostasis (Cohen et al., 2016). However, heightened and prolonged physiological responses to stress may signify ongoing mental health challenges. For example, acute psychological stress normatively increases production of inflammatory cytokines, such as Interleukin-6 (IL-6) (Marsland et al., 2017), but larger IL-6 increases are typically observed in those with more significant psychological ailments relative to less distressed individuals. Compared to their control counterparts, individuals with higher grief severity (Brown et al., 2022), higher depressive symptoms (Fagundes et al., 2013), or higher severity of loneliness (Jaremka et al., 2013) exhibited larger stress-induced elevations in IL-6. Exaggerated increases in stress-induced proinflammatory cytokine production may be problematic for long-term health, because such patterns were previously associated with higher basal levels of C-reactive protein, a biomarker of systemic inflammation (Lockwood et al., 2016), and changes in cardiometabolic health (Zannas et al., 2020), both conditions that are closely related to depression severity (Kiecolt-Glaser et al., 2015; Pan et al., 2012). In sum, existing work related to stress reactivity, particularly those pertaining to stress-induced cytokine production, suggest that the magnitude of the inflammatory response to stress not only identifies those experiencing *current* significant mental and physical health challenges but also those most likely to exhibit *future* symptoms.

Research related to psychological distress and physiological stress reactivity provide the groundwork for how emotional patterns and inflammatory response patterns may forecast future depressive symptom severity (Madison et al., 2022). A growing body of research suggests that psychological stress and stress reactivity patterns interactively predict future depressive symptom severity. For example, in a sample of healthy married adults, more frequent self-reported interpersonal stress at baseline was associated with higher depressive symptom severity at follow-up when participants also exhibited greater inflammatory responses to a marital conflict task at baseline (Madison et al., 2022). The interactive effect of psychological stress and biological stress patterns on depressive symptom severity may be understood within the diathesis-stress framework, which posits that mental disorders develop from a complex interaction between stress exposure and vulnerabilities (diathesis). The diathesis-stress model was developed to explain individual differences in psychopathology risk after stress exposure. Given their stability over time (Hassellund et al., 2010; Marsland et al., 2002; von Känel et al., 2006), stress reactivity patterns have been considered a vulnerability factor. Indeed, several studies have shown that neuroendocrine, autonomic, and inflammatory stress response patterns (diathesis) determined the strength of the relationship between stress exposure and mental health symptom severity (Madison et al., 2022; Obradović et al., 2010; McKeever and Huff, 2003; Eisenlohr-Moul et al., 2018; Daches et al., 2019). Notably, most pre-existing studies focused on either stress ratings or stressor count rather than affective ratings. Although stress is often associated with negative emotions such as anger, anxiety, fear, and guilt, some stressors may also evoke positively-valenced affect such as feeling inspired or determined (Lazarus, 2006). Thus, while stress and emotion are interrelated constructs, emotions provide additional information about how an individual is interacting with and adapting to their environment (Lazarus, 2006). Whether negative emotion patterns (conceptualized in the context of the diathesis-stress model as an index of sustained emotional distress) and physiological reactivity (conceptualized as a biological vulnerability or diathesis) synergistically predict future depressive symptom severity have yet to be explored. Given the role of negative affect in predicting depression prognosis and in bereavement coping, examining person-level factors under which negative affect may predict increases in depressive symptom may help identify maladjusted individuals.

In the present study, we examined whether stress-induced inflammatory responses and daily negative affect predict increases in depressive symptoms over time. Among bereaved spouses, we hypothesized that higher daily negative affect would be associated with an increase in depressive symptoms when individuals also exhibited larger IL-6 increases to a standardized laboratory stressor. As an exploratory aim, we examined group differences in these patterns by comparing bereaved participants to nonbereaved adults, hypothesizing that the above-mentioned associations would be more pronounced in bereaved participants compared to nonbereaved adults.

## 2. Methods and Materials

### 2.1. Study design and participant sample

Data for this study came from two study visits of *Project Heart*, a longitudinal observational study examining biobehavioral mechanisms underlying increased cardiovascular risk during the first year of spousal bereavement. Detailed recruitment methods and inclusion/exclusion criteria have been described previously (Fagundes et al., 2018). Briefly, bereaved adults were enrolled within three months post-loss and were married to their spouse for a minimum of three years. Nonbereaved adults were not required to be married/partnered. Exclusion criteria included the following: significant visual or auditory impairment, pregnant or nursing, diagnosed with an autoimmune or inflammatory disorder, divorced within the past year, and bereavement of another loved one in the past year. All participants provided written informed consent and study procedures were approved by Rice University Institutional Review Board.

As part of the parent study, participants underwent the Trier Social Stress Test (TSST) (Marsland et al., 2017; Kirschbaum et al., 1993) and a follow-up visit occurring eight months later. For bereaved adults, the TSST visit occurred at approximately 4 months post-loss, and the follow-up visit occurred at 12 months post-loss. At these in-person visits, venous blood samples, anthropometric measures, and self-reported health questionnaires were acquired in the laboratory. Immediately after the TSST visit, participants completed a 7-day daily diary probing daily affect and sleep behavior.

*Project Heart* participants who completed the TSST and provided a minimum of one valid pre-stressor blood draw were included in the present sample. From February 2016 to March 2020, 230 participants (151bereaved and 79 controls) completed a laboratory social stressor. Of the 230 participants, 18 were excluded from analyses either because blood draws were not administered or blood draw complications rendered the blood samples insufficient, resulting in a final sample size N = 212 (*n*_bereaved_ = 143, *n*_control_ = 69). Excluded participants did not differ statistically from the analytic sample on demographic (age, gender, race, education) and health-related (baseline BMI, comorbidity, anti-inflammatory medication use, depressive symptoms) characteristics. In this study, the TSST visit and follow-up visit are referred to as T1 and T2, respectively.

### 2.2. Measures

#### Stress-induced inflammatory response: IL-6 slope.

Stress-induced inflammatory response was quantified as the change in serum IL-6 over three time points during the Trier Social Stress Test (TSST), a standardized laboratory stressor known to induce reliable increases in proinflammatory cytokine production (Marsland et al., 2017; Kirschbaum et al., 1993). The TSST and immunoassay protocols for the parent study have been described previously (Brown et al., 2022; Wu-Chung et al., 2025). Briefly, participants underwent a standardized TSST protocol, in which they performed a public speaking task and a math task in front of two novel staff members in white laboratory coats. Blood was drawn approximately 10 min before the TSST (pre-TSST), 45 min post-TSST, and 120 min post-TSST. After the TSST, participants completed a 3-item survey assessing their perceived stress levels toward the a) speech portion, b) math portion, and c) task overall using a 7-point Likert scale with higher values indicating greater stress severity following the TSST (Mean across the 3 items = 4.96, SD = 1.31, median = 5). All TSST visits took place in the morning and self-report questionnaires were completed after the last blood draw. Serum aliquots were stored at −80 °C until assayed in duplicates for levels of IL-6 using high-sensitivity enzyme-linked immunosorbent assays (Quantikine, R&D Systems, Minneapolis, MN). The detection sensitivity of these assays was 0.16–10 pg/ml. Intra- and inter-assay coefficients of variation were 3.6–4.7% and 3.9–10.8%, respectively.

**Fig. 1.**
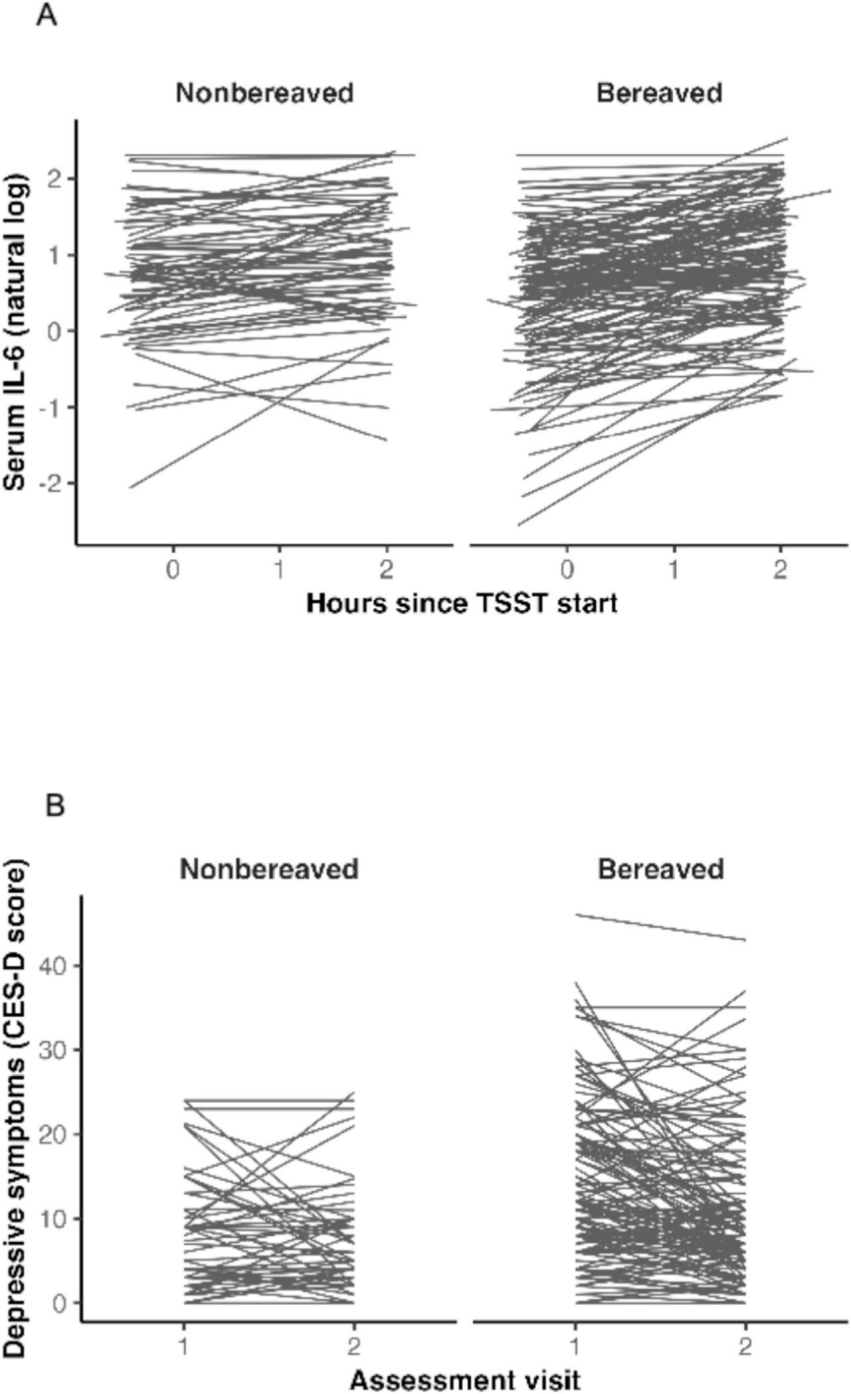
Spaghetti plots depicting A) IL-6 slopes (change in serum IL-6 in response to the TSST) and B) depressive symptoms from T1 to T2.

Because IL-6 values were positively skewed, they were natural log-transformed prior to all analyses. Linear mixed models evaluated natural log IL-6 as a function of continuous time (hours since start of TSST), with random intercepts for immunoassay batch and person, and a random slope for time within person. Analyses also evaluated, but failed to support, potential nonlinear (e.g., quadratic) effects of time. Therefore, the slope of time was taken to characterize change in IL-6 over time for each participant (see Fig. 1A for spaghetti plot of individual slopes). Maximum likelihood estimation was used to account for missing data. Slopes were extracted using the *ranef* function from the *lme4* package (Bates et al., 2022). No covariates were included in the model from which IL-6 slopes were extracted.

#### Depressive symptoms.

Depressive symptoms were measured using the *Center for Epidemiological Studies Depression Scale* (CES-D) (Radloff, 1977) at T1 and T2. Higher scores indicate more severe depressive symptoms and scores ≥ 16 indicate clinically significant depression. The reliability of the CES-D scale was high (α = 0.91).

#### Daily negative affect.

Participants self-reported up to 7 days of daily affect after the TSST visit (T1). Daily surveys were sent every morning before participants woke up. Five items from the *Positive and Negative Affect Scale* (PANAS) were used to measure negative affect (Watson et al., 1988). Participants answered based on the extent to which they *currently* felt this way. Items corresponding to negative affect included: ‘distressed’, ‘upset’, ‘afraid’, ‘nervous’, and ‘scared.’ Respondents rated each mood state using a 5-point scale ranging from 1 = very slightly or not at all to 5 = extremely. A summed total for each day was acquired (range: 5–25), with higher values indicating more negative affect. Person-level average across all days was used in analyses.

#### Covariates and descriptive variables.

Participants self-reported sociodemographic information (age, gender, race, ethnicity, and education) and medical history. Education was quantified as the highest grade (or year) of school one completed (Range: 1–20). Comorbid conditions were quantified using the Charlson Comorbidity Index, which designates weights to conditions based on their potential influence on one-year mortality (Charlson et al., 1994). Scores range from 0 to 37, with higher values indicating more severe comorbidity. Use of medications with anti-inflammatory properties (i.e, statins, metformin, non-steroidal anti-inflammatory drugs (NSAIDS), and COX-2 inhibitor drugs) was coded as a binary variable. Body mass index (BMI) at T1 was computed as weight in kilograms divided by height in meters squared.

### 2.3. Statistical analyses

All analyses were conducted in RStudio (Version 1.4.1106). For a list of packages, see supplemental material. We examined assumptions of linearity, normality, homoscedasticity, and multicollinearity (VIF) using diagnostic plots in R; all assumptions were met. Among the 212 participants who completed the TSST, approximately 69% (67% controls, 71% bereaved) completed at least one entry of the 7-day daily diary at Visit 2 (Mean: 6.03 days, SD = 1.50); 14% (n = 30) did not return for their last visit (Visit 4). To utilize all available data, full-information maximum likelihood (FIML) was employed to account for missing data. Full-information likelihood handles missing data better than listwise deletion and performs well, even with nonnormal data and small sample sizes (Shin et al., 2017). For transparency, we also ran sensitivity analyses using listwise deletion; these results can be found in supplemental material. Primary findings remained consistent across methods.

To examine the association between negative affect, TSST IL-6 slope, and change in depressive symptoms, we used SEM path analysis: T2 CES-D was regressed on T1 CES-D, TSST IL-6 slope (T1), daily negative affect, and the interaction between IL-6 slope and daily negative affect. Analyses evaluated this model with and without adjustment for covariates: age, gender, education, BMI, anti-inflammatory medication use, comorbidity index, and days between T1 and T2. In all models, negative affect and IL-6 slope were grand-mean centered prior to creating interaction variables. A significant interaction effect was examined further via simple slope testing using the probe2wayMC function in *semtools*: the conditional effects of the predictor on the outcome variable was evaluated at mean, +1 SD and −1 SD levels of the moderator (i.e., IL-6 slope). Because slopes reflected change in natural log transformed values of IL-6 (pg/mL) per hour, we provided the exponentiated interpretation to enhance interpretability of mean, +1 SD and −1 SD IL-6 slope values. That is, the exponentiated value represents the multiplicative change per hour. For example, when a natural log transformed IL-6 slope value of 0.11 is exponentiated (exp(0.11) = 1.116), this new value represents a multiplicative factor of 1.116, corresponding to an 11.6% increase in IL-6 per hour (Note: values below 1.0 would correspond to a decrease). For mathematical denotations, see supplemental material. To ensure the robustness of results, we conducted sensitivity tests 1) controlling for additional covariates (i.e., positive affect, antidepressant medication, grief severity, number of daily diary entries), 2) controlling for relationship satisfaction and marital duration, and 3) excluding influential outliers (see supplemental material); notably findings remained unchanged.

## 3. Results

### Descriptive and preliminary analyses.

Sample characteristics and bivariate correlations can be found in Table 1 and 2, respectively. At T1, 39% of bereaved spouses and 13% of nonbereaved adults had clinically significant depression levels (CESD ≥ 16). Bereaved participants also showed a larger decrease in CES-D over the 8-month period (Mean = −3.07, SD = 7.50) compared to nonbereaved participants (Mean = −0.54, SD = 6.32), *F*(1, 211) = 4.76, *p* = 0.031. Fig. 1B depicts change in CES-D for each subject; notably, increases and decreases in CES-D were observed in both groups. However, the variability in how depressive symptoms changed over the 8-month period was comparable between groups, as determined by a nonsignificant Levene’s test (*p* = 0.15). No significant group differences in daily negative affect were observed between bereaved and nonbereaved participants (see Table 1); variance in IL-6 slope (*p* = 0.09) and daily negative affect (*p* = 0.22) were also comparable across groups, despite bereaved individuals showing, on average, more positive IL-6 slopes than nonbereaved adults (*p* = 0.001, see Table 1 and Fig. 1A). Bivariate correlations revealed nonsignificant associations between daily negative affect and IL-6 slopes across the entire sample (*p* = 0.69).

### Primary aim.

**Fig. 2.**
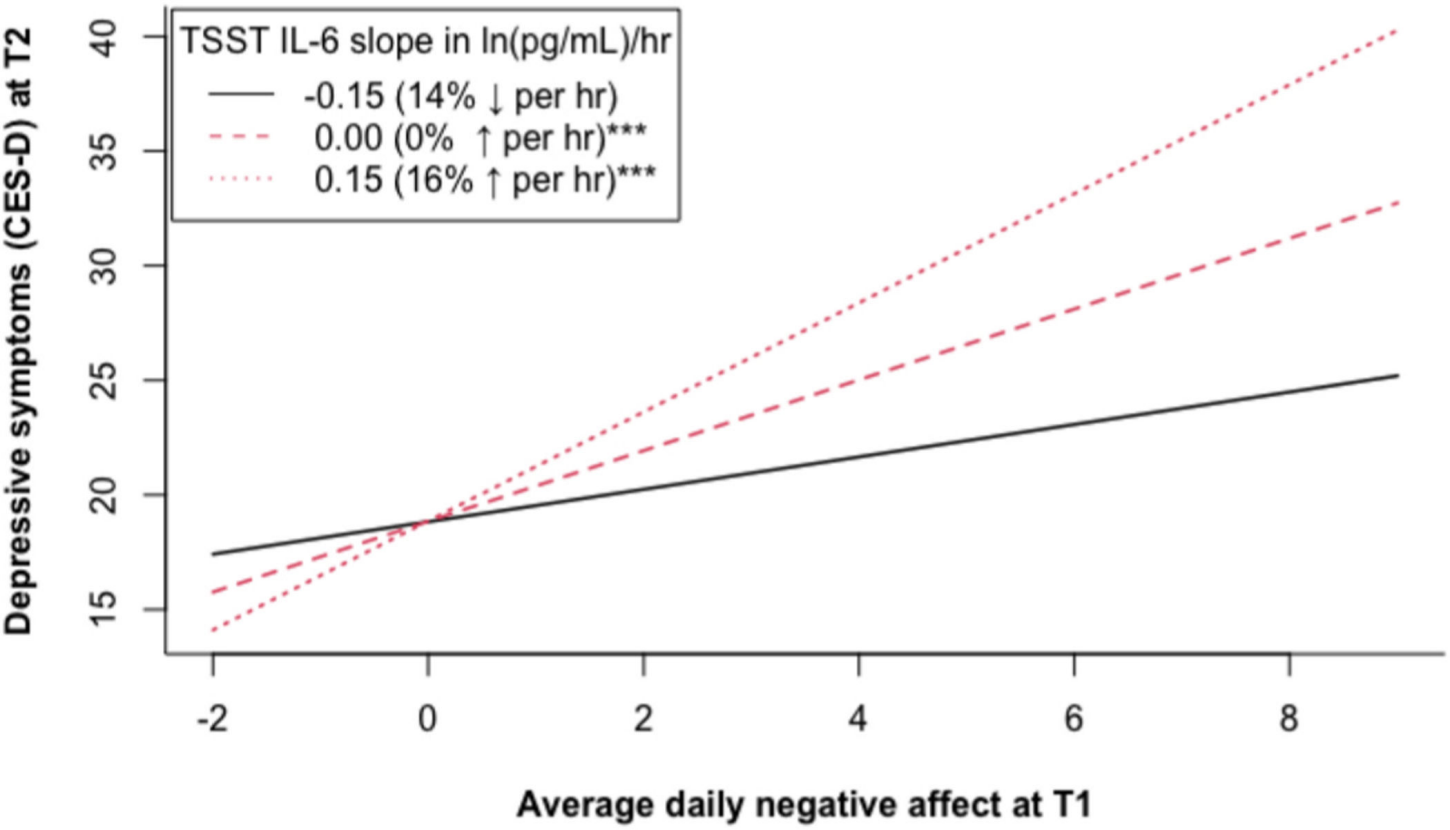
The association between T1 daily negative affect and T2 depressive symptoms at −1 SD, Mean, and + 1 SD of TSST IL-6 slope among bereaved spouses (N = 143). ***Note.*** Red lines indicate significant slopes. Negative affect and IL-6 slope were grand-mean centered. Models controlled for T1 depressive symptoms and covariates.

Among bereaved spouses, there was a statistically significant IL-6 slope × negative affect interaction effect on T2 depressive symptoms in both unadjusted (*p* = 0.029) and adjusted models (*p* = 0.018) (Table 3). The conditional effects of negative affect on depressive symptoms were explored at mean, +1 SD, and −1 SD values of grand-mean centered IL-6 slope which corresponded with a 0% increase per hour (0.00 ln pg/mL per hr), 16% increase per hour (0.15 ln pg/mL per hr), and a 14% decrease per hour (−0.15 ln pg/mL per hr), respectively. Examination of simple slopes revealed that negative affect was positively associated with future depressive symptoms only among individuals with average (*b* = 1.54, *SE* = 0.40, *p* < 0.001) or above-average (+1 SD) IL-6 slope (*b* = 2.38, *SE* = 0.55, *p* < 0.001), see Fig. 2. There was no association between negative affect and future depressive symptoms for bereaved individuals with below-average (−1 SD) IL-6 slope (*p* = 0.160).

### Exploratory aim.

**Fig. 3.**
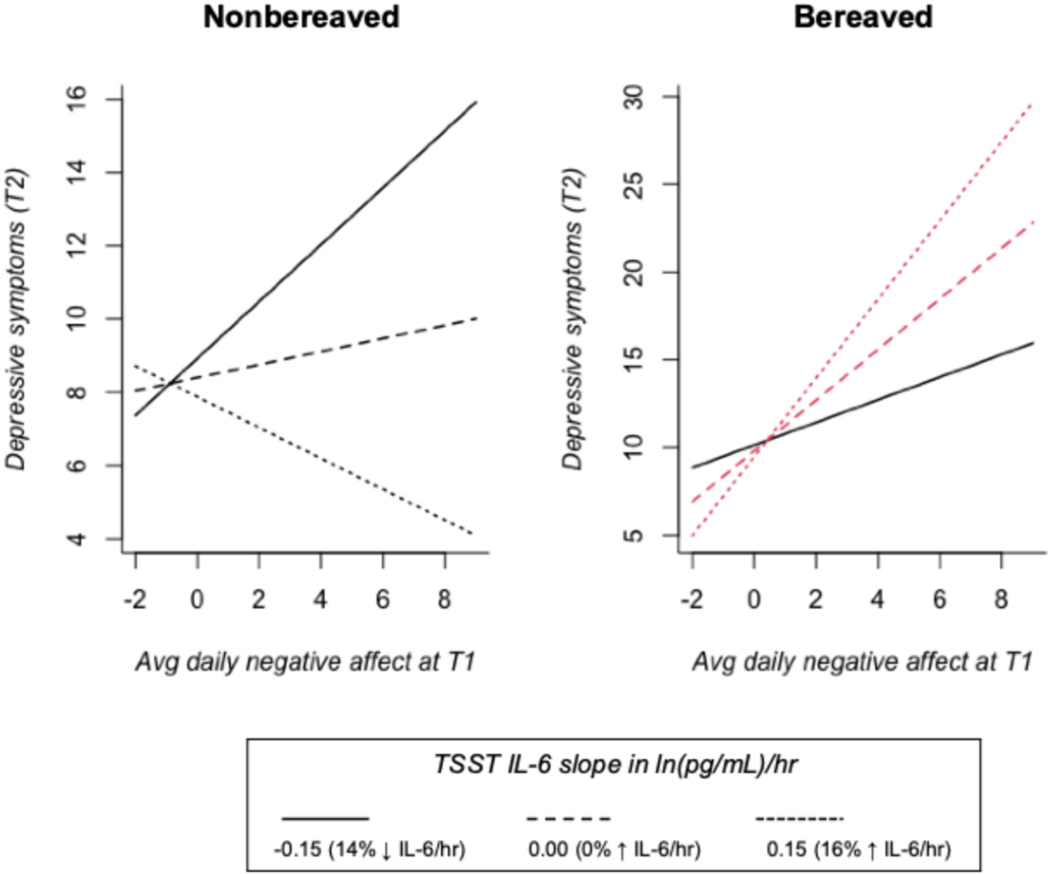
The association between T1 daily negative affect and T2 depressive symptoms at −1 SD, Mean, and + 1 SD of TSST IL-6 slope among bereaved and nonbereaved adults (N = 212). ***Note.*** Red lines indicate significant slopes. Negative affect and IL-6 slope were grand-mean centered. Models controlled for T1 depressive symptoms and covariates.

The 3-way interaction between IL and 6 slope, negative affect, and group (Table 4; Fig. 3) was then analyzed in unadjusted and adjusted models. Unadjusted (*p* = 0.011) and adjusted models (*p* = 0.010) supported the 3-way interaction effect on CES-D at T2, controlling for baseline CES-D (Table 4, Fig. 3). Examination of simple slopes revealed that negative affect was positively associated with T2 depressive symptoms, after accounting for T1 depressive symptoms, for bereaved participants showing average (*b* = 1.45, *SE* = 0.37, *p* < 0.001) and above-average (+1 SD) IL-6 responses to acute stress (*b* = 2.24, *SE* = 0.49, *p* < 0.001). There was no association between negative affect and depressive symptoms among bereaved participants with below-average (−1 SD) IL-6 responses to acute stress (*p* = 0.206). For control participants, there was no association between negative affect and depressive symptoms at any level of IL-6 slope (all *p*-values > 0.151).

## 4. Discussion

The present study examined the joint contributions of daily affect and IL-6 production to a standard stressor on future depressive symptoms in the context of a stressful life event (e.g., spousal bereavement). Descriptive analyses showed that the bereaved and nonbereaved group were comparable in demographic characteristics and various mental and physical health parameters at baseline, except depressive symptomology which was expectedly higher among bereaved individuals. Moreover, both groups showed similar variability in daily negative affect, IL-6 responsivity to stress, and 8-month change in depressive symptoms. Among recently bereaved spouses, IL-6 response patterns did modify the association between daily negative affect and future depressive symptoms, such that more overall negative affect in daily life was associated with a larger 8-month increase in depressive symptoms when bereaved spouses also exhibited an exaggerated IL-6 response to acute stress (e.g., sharper stress-induced increases in IL-6). Daily negative affect and future depressive symptoms were not associated for individuals who showed a decrease in IL-6 during an acute stressor. Additionally, there was preliminary evidence that the synergistic effect of daily negative affect and stress-induced IL-6 responses on future depressive symptoms was only pertinent within the context of a recent, major life stressor, as these patterns were not statistically significant among nonbereaved adults. This study makes novel contributions to the field, which has primarily focused on the cross-sectional (Fagundes et al., 2013; Brown et al., 2022; Jaremka et al., 2013; Wu-Chung et al., 2025; Kuhlman et al., 2022) rather than the longitudinal health implications of heightened inflammatory responses to stress.

Affective patterns during early bereavement provide valuable information about long-term psychological well-being (Coifman et al., 2007; Ong et al., 2004). Expectedly, during the first few months of bereavement, even the most resilient individuals can exhibit profound emotional distress and preoccupation with thoughts of the loss (Bonanno et al., 2004). Beginning at four months post-loss, individual differences start to emerge, making it a critical time point for assessing individual difference patterns (Bonanno, 2004; Bonanno and Kaltman, 2001). Consistent with known associations between affect and depression (Panaite et al., 2020), we found that negative affect – specifically emotions related to feeling distressed, upset, afraid, nervous and/or scared – was positively correlated with concurrent and future depressive symptoms. However, simply examining negative affect may yield an inaccurate prediction of adjustment, as a large majority of bereaved individuals still experiencing emotional upheaval after 4 months post-loss follow a normative grief trajectory, eventually exhibiting symptom abatement after one year post-loss (Bonanno et al., 2002). Guided by psychoneuroimmunology research demonstrating that exaggerated inflammatory responses to stress may alter the magnitude of the association between stress and depressive symptoms (Madison et al., 2022; Slavich and Irwin, 2014), we provide some of the first evidence that, within the context of bereavement, one’s affective and inflammatory patterns may be an early proxy of future depressive symptom severity. In particular, bereaved spouses who experienced greater negative emotion in daily life were more likely to show an 8-month increase in depressive symptoms, especially if they also exhibited a more exaggerated IL-6 response to acute, social stress. These findings suggest that while more negative emotions within the first few months of bereavement do predict an increase in depressive symptoms, bereaved spouses that mount a larger biological response to social stress may show the largest increase in symptoms over time.

Although inflammatory increases to acute stress are normal, exaggerated increases in stress-induced inflammation may reflect a neuroimmune circuit sensitive to social-environmental challenges and skewed toward a proinflammatory phenotype (Slavich and Irwin, 2014; Eisenberger and Cole, 2012; Slavich and Cole, 2013; Eisenberger et al., 2017). This neuroimmune circuitry consists of brain regions (e.g., dorsal anterior cingulate cortex, amygdala, anterior insula) involved in threat- and negative affect processing that relay information to the hypothalamus (Eisenberger and Cole, 2012). The hypothalamus directly regulates stress response pathways that, in turn, modulate inflammatory activity. Feedback pathways allow inflammatory mediators to modulate the same brain regions initially processing the threat, such as those affecting perceptions of threat appraisal. Thus, bidirectional neuroimmune pathways enable top-down regulation of inflammatory signaling and bottom-up immune-mediated regulation of cognitive, affective, and somatic processes. Over months and years, a sensitized psychobiological pathway that is highly attuned to real or perceived adversity and biased toward proinflammatory signaling may increase susceptibility to inflammation-related conditions. Indeed, heightened inflammatory responses to psychological stress have previously been associated with higher resting levels of CRP, a biomarker of systemic inflammation (Lockwood et al., 2016). Notably, the inflammatory response to psychological stress varies substantially across individuals and remains stable across tasks and repeated assessments (Marsland et al., 2002; von Känel et al., 2006), substantiating the possibility that the magnitude of this response may indicate vulnerability to inflammation-related conditions following stress encounters.

A neuroimmune pathway sensitized to adversity is central to existing theory linking social threats such as rejection, loss, and isolation to depression onset and progression (Slavich and Irwin, 2014; Nusslock et al., 2024). Although elevated levels of peripheral inflammatory biomarkers are commonly observed among those with depression, few studies have explored whether the immune system’s responsiveness to psychological stress predicts future depressive symptoms. Madison et al. (2022) showed that social threats (e.g., perceived social isolation, interpersonal stress) increased depressive symptoms over time and such increases were only observed when individuals showed a larger increase in IL-6 toward a standardized social stressor. Tangentially, we showed that negative affect experienced within the context of profound social loss was associated with an increase in depressive symptoms, especially when bereaved spouses showed a larger increase in IL-6 to a standardized social stressor. Altogether, existing evidence suggests that inflammatory response patterns may be a valuable individual difference factor for identifying which individuals are most susceptible to worsening depressive symptoms and, thus, may require additional therapeutic attention.

Exploratory analyses revealed that stress-induced IL-6 response patterns only moderated the association between daily negative affect and future depressive symptoms for individuals who recently experienced spousal loss. Although our sample size was too small to draw definitive conclusions about this association, the Stress Sensitization model may provide insight into these findings. The Stress Sensitization model posits that successive experiences of stress decrease the threshold for episodes of affective disorders (Stroud et al., 2020). In the context of spousal bereavement within this model, individuals experiencing repeated stress in the form of consistent grief, rapid change in daily responsibilities, and major social shifts may have a lower threshold for experiences of depressive symptoms (Stroebe and Schut, 1999). As such, biopsychosocial risk factors (i.e., daily negative affect and inflammatory response to stress) may fuel depressive symptoms among people experiencing major life stress, while individuals without major life stress remain resilient to depression despite similar biopsychosocial risk. Future research should aim to replicate these results with larger samples and in other stressful contexts to clarify the circumstances under which high negative affect and stress-induced inflammatory responses may be particularly informative for understanding depression risk and progression.

This study adopted an innovative methodological design that lends several strengths. First, multi-day reports of affect provide more accurate information about a person’s day-to-day emotional experience than one-time retrospective reports, which are influenced by peak affect and memory bias toward negative experiences (Ganzach and Yaor, 2019). By using daily diaries, we can better understand how daily processes contribute to the development or progression of depressive symptoms after exposure to a major life stressor. Further, we quantified physiological responses to a well-validated experimental stressor. The coupling of laboratory measures with daily self-report provides a strong foundation for understanding how biopsychosocial patterns predict future depressive symptoms.

These findings carry limitations that provide directions for future research. Primarily, the sample’s racial, ethnic, and socioeconomic diversity limits the generalizability of these findings. Similarly, although bereavement is among life’s most stressful experiences (Holmes and Rahe, 1967), it is unclear whether these findings would be replicated in a different model of major life stress (e.g., caregiving, divorce, etc.). As such, future research should include a racially and socioeconomically diverse sample to enhance the potential impact of these findings, and future designs could investigate the roles of other major life stressors. Further, capturing mean levels of daily negative affect neglects the dynamic nature of emotional processes, which may be relevant for understanding depression severity. Emotions are dynamic processes that can vary within minutes (Koval et al., 2021), and relying on a single affective measure per day only provides a snapshot of the emotional processes that characterize bereavement and future depressive symptoms. Additionally, observed patterns may reflect high-arousal negative emotion specifically, as daily self-report measures did not assess low-arousal states more commonly experienced in bereavement, such as sadness and lethargy. Future studies that examine a wider spectrum of negative affect may help clarify whether observed patterns can be generalized to both high- and low-arousal negative affect. Lastly, group characteristics both strengthen and temper interpretations of the 3-way interaction. While the focus on daily emotions less specific to bereavement may have contributed to null differences in baseline daily negative affect between bereaved and nonbereaved adults, comparable levels of high-arousal negative affect across groups ensured that interaction effects were not driven by differences in bereavement-specific negative affect. At the same time, less change in depressive symptoms in the nonbereaved group may have limited our ability to detect similar moderating effects of stress-induced IL-6 responses in that group. These considerations, in addition to accommodating a sufficient sample size for testing 3-way interactions, should be weighed in the design of future replication studies.

Individual differences in how people respond to their environment may account for variations in long-term mental well-being following stressful life event exposure. Among bereaved spouses, we found that those who experienced more negative affect in their daily life showed an increase in depressive symptom severity over time, and this increase was largest for those who showed greater IL-6 production to acute stress. In sum, this study identified novel psychobiological patterns predictive of future maladjustment to spousal bereavement and further substantiated the possibility that inflammatory response patterns may carry clinical significance, especially with regard to stress-related disease risk.

## Supplementary Material

Supplementary Material

## Figures and Tables

**Table 1:** Sample characteristics and variables of interest.

	Nonbereaved (N = 69)	Bereaved (N = 143)	Total (N = 212)	*p* value
Age	68.28 (13.11)	68.21 (8.79)	68.23 (10.36)	0.96
Gender (Female)	51 (73.91%)	92 (64.34%)	143 (67.45%)	0.163
Body mass index	28.85 (6.64)	27.63 (5.29)	28.03 (5.77)	0.148
Race				**0.027**
*White*	50 (72.46%)	127 (88.81%)	177 (83.49%)	
*Black/African American*	12 (17.39%)	9 (6.29%)	21 (9.91%)	
*Asian*	3 (4.35%)	3 (2.10%)	6 (2.83%)	
*Other*	4 (5.80%)	4 (2.80%)	8 (3.77%)	
Comorbid conditions	0.45 (2.78)	0.36 (1.00)	0.39 (1.78)	0.726
Medication use (yes)	33 (47.83%)	70 (48.95%)	103 (48.58%)	0.878
Education	15.93 (2.45)	16.06 (2.61)	16.01 (2.55)	0.956
Daily negative affect	5.91 (1.55)	6.46 (1.92)	6.29 (1.82)	0.106
Daily positive affect	16.22 (4.18)	14.12 (4.07)	14.78 (4.21)	**0.005**
Days between T1 and T2	229.87 (28.90)	232.40 (24.92)	231.64 (26.13)	0.550
CES-D (T1)	7.77 (7.16)	14.34 (9.74)	12.20 (9.48)	**< 0.001**
CES-D (T2)	7.18 (6.45)	10.95 (8.65)	9.81 (8.22)	**0.004**
IL-6 slope	−0.05 (0.13)	0.02 (0.15)	−0.00 (0.15)	**0.001**

*Note:* Medication use involves taking the following medication: statins, metformin, non-steroidal anti-inflammatory drugs (NSAIDS), COX-2 inhibitor drugs, and other anti-inflammatory medication (i.e., immunosuppressants). T1 = Trier Social Stress Test visit, T2 = 8 months since TSST visit; IL-6 slope is the change in natural log transformed IL-6 (pg/mL) over time across 3 time points (10 min before TSST, 45 min post-TSST, 120 min post-TSST).

**Table 2:** Bivariate Pearson correlations of main variables of interest.

Variable	1	2	3	4	5	6	7	8	9	10
1. IL-6 slope										
2. CES-D (T1)	0.21**									
3. CES-D (T2)	0.05	0.66**								
4. Negative affect	0.03	0.57**	0.54**							
5. Positive affect	−0.07	−0.54**	−0.42**	−0.36**						
6. Age	−0.22**	−0.17*	−0.10	−0.15	0.24**					
7. Female	0.28**	0.11	0.09	0.13	−0.11	−0.09				
8. BMI	−0.16*	0.07	0.12	−0.10	0.03	−0.06	−0.09			
9. Education (yrs)	−0.02	−0.10	−0.11	0.14	−0.07	−0.04	−0.13	−0.23**		
10. Comorbidity	−0.11	0.04	0.04	−0.09	0.09	0.12	0.03	0.02	−0.05	
11. Perceived stress of TSST	−0.05	0.18*	0.13	0.02	−0.15	−0.08	0.19**	0.05	−0.23**	0.11

Note.

* indicates *p* < 0.05.

** indicates *p* < 0.01. T1 = Trier Social Stress Test (TSST) visit. T2 = 8 months since TSST visit. CESD = Center for Epidemiological Studies Depression Scale (higher scores indicate more severe depressive symptoms). Average daily negative and positive affect were assessed using 10 items from the Positive and Negative Affect Scale (higher scores indicate more negative/positive affect). IL-6 slope is the change in natural log transformed serum IL-6 (pg/mL) per hour; slopes were extracted from mixed level models that modeled natural log IL-6 across 3 time points (approximately 10 min before TSST, 45 min post-TSST, 120 min post-TSST). Comorbidity is the total score on the Charlson Comorbidity Index. Perceived stress was an average of 3 items that probed subjects’ level of stress towards the a) speech portion, b) math portion, and c) the TSST task overall on a 7-pt Likert scale (range: 1–7), with higher values indicating more stress.

**Table 3:** Depressive symptoms at follow-up regressed on TSST IL-6 slope, daily negative affect, and covariates at Time 1 (N = 143).

	CES-D at Time 2 (8-month follow-up)							
Predictor	Estimate	SE	p	CI	Estimate	SE	p	CI
TSST IL-6 slope^a^	−2.14	3.66	0.559	−9.30 – 5.03	0.11	4.09	0.978	−7.90 – 8.13
Negative affect^a^	1.39	0.38	**< 0.001**	0.64 – 2.14	1.54	0.40	**< 0.001**	0.77 – 2.32
TSST IL-6 slope × Negative^a^ affect	5.03	2.30	**0.029**	0.51 – 9.54	5.39	2.29	**0.018**	0.91 – 9.87
CES-D at Time 1	0.44	0.07	**< 0.001**	0.30 – 0.59	0.43	0.07	**< 0.001**	0.29 – 0.57
Age					−0.07	0.07	0.362	−0.21 – 0.08
Gender [Female]					−0.24	1.22	0.844	−2.63 – 2.15
BMI					0.09	0.10	0.414	−0.12 – 0.29
Medication use					0.65	1.20	0.584	−1.69 – 3.00
Comorbidity					0.59	0.53	0.267	−0.45 – 1.62
Education (yrs)					−0.54	0.22	**0.014**	−0.98 – −0.11
Days between visits					−0.02	0.02	0.483	−0.06 – 0.03

Note.

a Variables were grand-mean centered. TSST IL-6 slope = change in natural log transformed serum IL-6 (pg/mL) in response to the Trier Social Stress Test administered at Time 1; Negative affect was assessed using negative affect subscale of PANAS, CES-D = Center for Epidemiological Studies Depression Scale, BMI = body mass index; Medication use and gender were coded as binary. Medication use consisted of statins, metformin, non-steroidal anti-inflammatory drugs (NSAIDS), and COX-2 inhibitor drugs.

**Table 4:** Depressive symptoms regressed on daily negative affect, IL-6 slope, bereavement status, and covariates (N = 212).

	CES-D at Time 2 (8-month follow-up)							
Predictors	*Estimate*	*SE*	*p*	*CI*	*Estimate*	*SE*	*p*	*CI*
TSST IL-6 slope^a^	−1.61	6.30	0.799	−13.96 – 10.74	−3.49	6.34	0.582	−15.92 – 8.94
Negative affect^a^	0.08	0.56	0.899	−1.03 – 1.18	0.18	0.57	0.755	−0.94 – 1.30
Group [Bereaved]	1.00	1.08	0.354	−1.11 – 3.11	1.41	1.10	0.200	−0.75 – 3.57
TSST IL-6 slope^a^ × Negative affect^a^	−4.18	2.88	0.147	−9.82 – 1.46	−3.99	2.88	0.166	−9.65 – 1.66
TSST IL-6 slope^a^ × Group	−1.34	7.20	0.853	−15.46 – 12.78	1.28	7.28	0.860	−12.98 – 15.55
Negative affect^a^ × Group	1.26	0.63	**0.045**	0.03 – 2.48	1.27	0.63	**0.043**	0.04 – 2.49
TSST IL-6^a^ × Negative affect^a^ × Group	9.27	3.63	**0.011**	2.15 – 16.39	9.32	3.64	**0.010**	2.19 – 16.46
CES-D at Time 1	0.44	0.06	**< 0.001**	0.32 – 0.56	0.41	0.06	**< 0.001**	0.29 – 0.54
Age					−0.04	0.05	0.387	−0.14 – 0.05
Gender [Female]					0.17	1.01	0.870	−1.81 – 2.14
BMI					0.11	0.08	0.158	−0.04 – 0.27
Medication use					0.24	0.92	0.795	−1.56 – 2.04
Comorbidity					0.46	0.45	0.309	−0.43 – 1.35
Education					−0.32	0.18	0.086	−0.68 – 0.04
Days between visits					−0.00	0.02	0.930	−0.03 – 0.03

Note.

a Variables were grand-mean centered. TSST IL-6 slope = change in natural log transformed serum IL-6 (pg/mL) in response to the Trier Social Stress Test administered at Time 1; Negative affect was assessed using negative affect subscale of PANAS, CES-D = Center for Epidemiological Studies Depression Scale, BMI = body mass index; Medication use and gender were coded as binary. Medication use consisted of statins, metformin, non-steroidal anti-inflammatory drugs (NSAIDS), and COX-2 inhibitor drugs. Group = Bereaved (1) or Nonbereaved (0).

## Data Availability

Data will be made available on request.

## Appendix A. Supplementary data

Supplementary data to this article can be found online at https://doi.org/10.1016/j.bbi.2026.106774.
