# Supplementary material for: Stress-induced IL-6 response patterns amplify the link between daily negative affect and later depressive symptoms during bereavement
Source: Brain Behav Immun. Author manuscript; Available in PMC 2026 Jul 20. (PMC13383662; doi:10.1016/j.bbi.2026.106774)
Supplement: Supplementary Material [file NIHMS2174349-supplement-Supplementary_Material.docx]

Supplemental Material for Wu-Chung et al.

Table of Contents

[List of R packages and citations 2](#_Toc222327410)

[Interpretating IL-6 slope that is based on natural log transformed values of IL-6: 4](#_Toc222327411)

[Note on PANAS 5](#_Toc222327412)

[Figure S1. Depressive symptoms at Time 2 (8 month follow-up) regressed on negative affect and inflammatory stress reactivity at Time 1, after controlling for depressive symptoms at Time 1 and relevant covariates. 6](#_Toc222327413)

[Table S1. Regress change results among bereaved spouses using listwise deletion 7](#_Toc222327414)

[Table S2. Regress change results from models using listwise deletion, testing 3-way interaction among bereaved and nonbereaved adults 8](#_Toc222327415)

[Sensitivity analyses: Additional Covariates 9](#_Toc222327416)

[Sensitivity analyses: Outliers 10](#_Toc222327417)

# **List of R packages and citations**

We used the following packages for data visualization, tables, and analyses: *lavaan* (1), *arsenal* (2), *apaTables* (3), *semTools* (4), *emmeans* (5), *stargazer* (6), influence.SEM (7), ggplot2 (8), sjPlot (9), rvg (10), officer (11), dplyr (12).

1. Rosseel Y. lavaan: An R package for structural equation modeling. Journal of Statistical Software. 2012;48(2):1–36.

2. Heinzen E, Sinnwell J, Atkinson E, Gunderson T, Dougherty G, Votruba P, et al. arsenal: An Arsenal of “R” Functions for Large-Scale Statistical Summaries [Internet]. 2021 [cited 2022 July 19]. Available from: https://CRAN.R-project.org/package=arsenal

3. Stanley D. apaTables: Create American Psychological Association (APA) Style Tables [Internet]. 2020. Available from: https://github.com/dstanley4/apaTables

4. Jorgensen TD, Pornprasertmanit S, Schoemann AM, Rosseel Y, Miller P, Quick C, et al. semTools: Useful Tools for Structural Equation Modeling [Internet]. 2025 [cited 2025 June 27]. Available from: https://cran.r-project.org/web/packages/semTools/index.html

5. Lenth RV. emmeans: Estimated Marginal Means, aka Least-Squares Means [Internet]. 2022. Available from: https://github.com/rvlenth/emmeans

6. Hlavac M. stargazer: Well-Formatted Regression and Summary Statistics Tables [Internet]. 2022 [cited 2025 June 27]. Available from: https://cran.r-project.org/web/packages/stargazer/index.html

7. Pastore M, Altoe G. influence.SEM: Case Influence in Structural Equation Models [Internet]. 2025 [cited 2025 Nov 13]. Available from: https://cran.r-project.org/web/packages/influence.SEM/index.html

8. Wickham H. ggplot2: Elegant graphics for data analysis [Internet]. Springer-Verlag New York; 2016. Available from: https://ggplot2.tidyverse.org

9. Lüdecke D. sjPlot: Data visualization for statistics in social science [Internet]. 2021. Available from: https://CRAN.R-project.org/package=sjPlot

10. Gohel D, Rudis B, Brunetti F. rvg: R Graphics Devices for Vector Graphics Output [Internet]. 2020 [cited 2022 Sept 13]. Available from: https://CRAN.R-project.org/package=rvg

11. Gohel D. officer: Manipulation of Microsoft Word and PowerPoint Documents [Internet]. 2022. Available from: https://davidgohel.github.io/officer/

12. Wickham H, François R, Henry L, Müller K. dplyr: A Grammar of Data Manipulation. [Internet]. 2020. Available from: https://CRAN.R-project.org/package=dplyr

13. Dejonckheere E, Mestdagh M, Houben M, Rutten I, Sels L, Kuppens P, et al. Complex affect dynamics add limited information to the prediction of psychological well-being. Nat Hum Behav. 2019 May;3(5):478–91.

14. Panaite V, Rottenberg J, Bylsma LM. Daily Affective Dynamics Predict Depression Symptom Trajectories Among Adults with Major and Minor Depression. Affec Sci. 2020 Sept 1;1(3):186–98.

.

# **Interpretating IL-6 slope that is based on natural log transformed values of IL-6:**

1. Converting slope in units of ln(pg/mL) per hour to percentage change per hour via exponentiation:

% change per hour = (e^β1^_​−_1) × 100%

β_1:_  IL-6 slope in units of ln(pg/mL) per hour.

*Example:* Convert IL-6 slope of 0.11 to % change per hour.

- % change per hour = (e^0.11^_​−_1) × 100%
- % change per hour = 11.63
- **Interpretation:** A IL-6 slope of 0.11 corresponds to a 11.63% increase in IL-6 per hour.

1. Converting slope in units of ln(pg/mL0 per hour to approximate change in pg/mL/hr at the sample median 2.5 pg/mL:

pg/ml/hr = Y_0_  × (e^β1^_​−_1)

β_1:_  IL-6 slope in units of ln(pg/mL) per hour.

Y_0_: median value of IL-6 (pg/mL) of the sample

Note: This is only valid near the chosen Y_0._ The absolute rate changes as IL-6 slope increases.

*Example:* Convert IL-6 slope of 0.11 to approximate change in pg/mL per hour.

- pg/ml/hr = 2.5 × (e^0.11^_​−_1)
- pg/ml/hr = 0.29
- **Interpretation:** A 11.6% increase in IL-6 per hour corresponds with an IL-6 increase of 0.29 pg/mL per hour at the sample median (2.5 pg/mL).

# **Note on PANAS**

On average, participants completed 4.03 out of 7 days of daily affect surveys. We examined average daily negative because we had few data points to reliably capture more complex metrics; moreover, prior work has shown that other dynamic measures such as standard deviation do not uniquely predict psychological outcomes such as depression above and beyond average negative and positive affect (13,14). Positive affect was not part of primary analyses; however, positive affect was included as a covariate in sensitivity analyses to ensure that observed patterns were not driven by low positive affect, which is a hallmark characteristic of depression.

# **Figure S1.** Depressive symptoms at Time 2 (8 month follow-up) regressed on negative affect and inflammatory stress reactivity at Time 1, after controlling for depressive symptoms at Time 1 and relevant covariates.


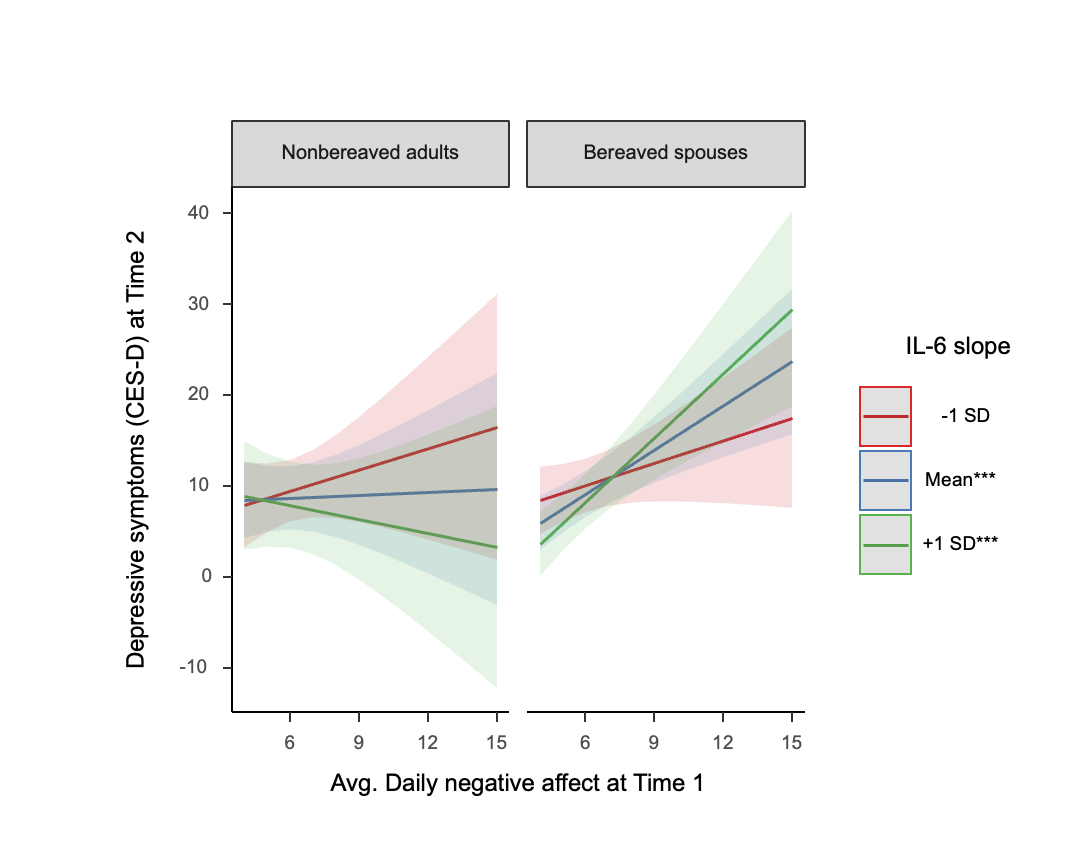


Note. Patterns reflect regress change models using listwise deletion.

# ***Table S1.*** *Regress change results among bereaved spouses using listwise deletion*

|  | CES-D at Time 2 (8-month follow-up) | | | | | |
| --- | --- | --- | --- | --- | --- | --- |
| Predictors | Estimates | CI | p | Estimates | CI | p |
| (Intercept) | 4.74 | 1.94 – 7.55 | **0.001** | **13.46** | -11.58 – 38.51 | 0.288 |
| TSST IL-6 slope^a^ | -4.34 | -14.36 – 5.68 | 0.392 | -3.98 | -16.23 – 8.27 | 0.519 |
| Negative affect^a^ | 1.47 | 0.62 – 2.31 | **0.001** | 1.61 | 0.69 – 2.53 | **0.001** |
| CES-D Time 1 | 0.46 | 0.28 – 0.63 | **<0.001** | 0.43 | 0.25 – 0.62 | **<0.001** |
| TSST IL-6 slope^a^ × Negative affect^a^ | 5.23 | 0.18 – 10.28 | **0.043** | 5.44 | 0.17 – 10.71 | **0.043** |
| Age |  |  |  | -0.10 | -0.32 – 0.13 | 0.394 |
| Gender [Female] |  |  |  | 0.18 | -3.08 – 3.44 | 0.912 |
| BMI |  |  |  | 0.05 | -0.23 – 0.34 | 0.720 |
| Comorbidity |  |  |  | 1.24 | -0.30 – 2.78 | 0.113 |
| Anti-inflammatory medication use [yes] |  |  |  | 0.13 | -2.98 – 3.23 | 0.936 |
| Education |  |  |  | -0.33 | -0.92 – 0.26 | 0.274 |
| Days between visits |  |  |  | 0.01 | -0.05 – 0.06 | 0.832 |
| Observations | 88 | | | 88 | | |
| R^2^ / R^2^ adjusted | 0.543 / 0.521 | | | 0.571 / 0.509 | | |

Note. ^a^Main variables of interest (negative affect, IL-6 slope) were grand mean-centered.

# ***Table S2.*** *Regress change results from models using listwise deletion, testing 3-way interaction among bereaved and nonbereaved adults*

|  | CES-D at Time 2 (8-month follow-up) | | |
| --- | --- | --- | --- |
| Predictors | Estimates | CI | p |
| (Intercept) | 2.28 | -15.98 – 20.53 | 0.805 |
| TSST IL-6 slope | -7.07 | -21.85 – 7.70 | 0.345 |
| Negative affect | 0.16 | -1.10 – 1.42 | 0.802 |
| Group [Bereaved] | 0.99 | -1.80 – 3.79 | 0.483 |
| CES-D Time 1 | 0.41 | 0.26 – 0.57 | **<0.001** |
| Age | -0.06 | -0.21 – 0.09 | 0.446 |
| Gender [Female] | 0.63 | -1.99 – 3.25 | 0.634 |
| BMI | 0.13 | -0.09 – 0.36 | 0.231 |
| Education | -0.10 | -0.59 – 0.39 | 0.687 |
| Days between visits | 0.01 | -0.03 – 0.05 | 0.520 |
| Comorbidity | 1.21 | -0.17 – 2.59 | 0.086 |
| Anti-inflammatory medication use [yes] | -0.10 | -2.41 – 2.20 | 0.929 |
| TSST IL-6 slope × Negative affect | -4.25 | -10.64 – 2.14 | 0.190 |
| TSST IL-6 slope × Group [Bereaved] | 2.65 | -15.22 – 20.52 | 0.769 |
| Negative affect × Group [Bereaved] | 1.38 | -0.01 – 2.77 | 0.051 |
| Negative affect × Group [Bereaved] × TSST IL-6 slope | 9.58 | 1.47 – 17.69 | **0.021** |
| Observations | 127 | | |
| R^2^ / R^2^ adjusted | 0.557 / 0.498 | | |
| *Note:* negative affect and IL-6 were grand mean centered | | | |

# **Sensitivity analyses:** Additional Covariates

Table S4. Estimates for interaction effect (negative affect × IL-6 slope) after controlling for extra covariates in models predicting CESD T2 in bereaved adults (N=143)

|  | Estimates for interaction effect (negative affect × IL-6 slope) | | |
| --- | --- | --- | --- |
| Covariates included in model | *b* | *p* | *CI* |
| Model 1: Base covariates | 5.39 | .018 | [.91, 9.87] |
| Model 2: Base + grief severity at T1 | 5.36 | .021 | [.82, 9.90] |
| Model 3: Base + days since passing | 5.41 | .019 | [.90, 9.92] |
| Model 4: Base + number of daily diary entries | 5.32 | .021 | [.80, 9.83] |
| Model 5: Base +daily positive affect | 5.45 | .019 | [.89, 10.01] |
| Model 6: Base + all covariates in Model 1-5 | 5.38 | .022 | [.79, 9.96] |
| Model 7: Base covariates + marriage length + marital satisfaction | 5.41 | .018 | [.94, 9.89] |
| *Note:* base covariates included age, gender, BMI, anti-inflammatory medication use, comorbidity, education (yrs), days between baseline and follow-up; these statistics were derived from path analysis in SEM using FIML to account for missing data. Marriage length (Mean: 37.13 yrs, SD = 14.71) was modeled continuously. Marital satisfaction (Mean = 4.13, SD = .96) was assessed with the following item and scoring parameters: “In general, how satisfied are you with your relationship with your spouse/partner?”; 0 = not at all, 1 = a little, 2 = somewhat, 3 = mostly, 4 = almost completely, 5 = completely. | | | |

# **Sensitivity analyses:** Outliers

We used Cook’s distance to identify influential outliers in the primary model of bereaved subjects. Models were rerun after excluding datapoints with a Cook’s distance greater than 1. Three influential outliers were identified (see plot). After excluding those participants, findings remained unchanged.


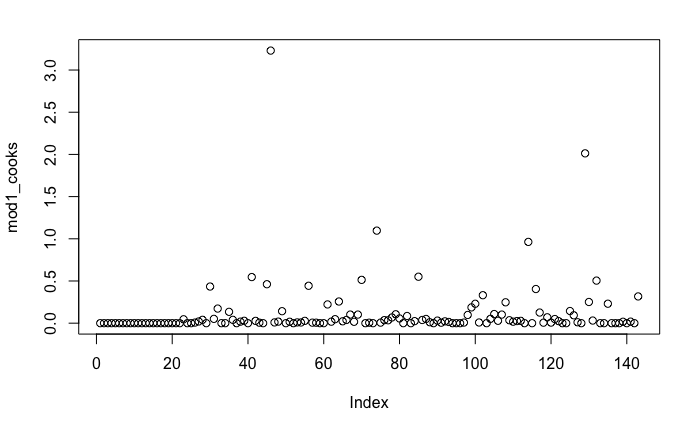


Table S5. Estimates for interaction effect (negative affect × IL-6 slope) after controlling for extra covariates in models predicting CESD T2 in bereaved adults (N=143)

|  | Estimates for interaction effect (negative affect × IL-6 slope) | | |
| --- | --- | --- | --- |
| Covariates included in model | *b* | *p* | *CI* |
| Model 1: Base covariates | 5.39 | .018 | [.91, 9.87] |
| Model 2: After excluding 3 outliers | 5.43 | .011 | [1.27 9.59] |

Note: These statistics were based on path analysis in SEM using FIML to account for missing data
